# QTL mapping for target leaf spot resistance in a Japanese-type cucumber

**DOI:** 10.1270/jsbbs.26005

**Published:** 2026-07-02

**Authors:** Minh Thi Ho, Ruikun Chen, Sachiko Isobe, Kenta Shirasawa, Yosuke Yoshioka

**Affiliations:** 1 Graduate School of Science and Technology, University of Tsukuba, 1-1-1 Tennodai, Tsukuba, Ibaraki 305-8572, Japan; 2 Institute of Life and Environmental Sciences, University of Tsukuba, 1-1-1 Tennodai, Tsukuba, Ibaraki 305-8572, Japan; 3 Kazusa DNA Research Institute, 2-6-7 Kazusa-kamatari, Kisarazu, Chiba 292-0818, Japan

**Keywords:** target leaf spot resistance, high-resolution melting markers, quantitative inheritance, disease severity

## Abstract

Target leaf spot (TLS), caused by *Corynespora cassiicola*, is among the most economically important diseases of cucumber (*Cucumis sativus* L.). Because of the rising incidence of disease problems, much effort has been put toward resistance breeding. Here, we investigated the inheritance of high TLS resistance in a Japanese–type inbred line. QTL analyses were performed using F_2_ and F_2:3_ families derived from a cross between the resistant and a susceptible (Beit Alpha–type) inbred line. We evaluated disease severity in F_2:3_ families in four independent assays and mapped a total of five TLS-resistance QTLs on chromosome (Chr.) 2, Chr. 5, and Chr. 6. The effect of the two major QTLs on Chr.5, *Tls5.1* and *Tls5.2*, was confirmed through additional assays and verified in different genetic backgrounds. Fine mapping using recombinants in several generations narrowed down *Tls5.2* to a 496.8-kb region with 69 genes, several of which are potential candidates involved in plant disease resistance. The newly identified resistance loci may confer more durable resistance when combined with previously characterized resistance genes or loci. Furthermore, molecular markers linked to TLS resistance derived from the Japanese–type inbred line could be effectively used to accelerate breeding for this trait.

## Introduction

Target leaf spot (TLS) is one of the most economically important foliar diseases in cucumber (*Cucumis sativus* L.) worldwide. This disease is caused by *Corynespora cassiicola* (Berk. & Curt) Wei, an imperfect ascomycete fungus (MacKenzie *et al.* 2018, Schlub *et al.* 2009), with severe infections leading to substantial yield loss. The pathogen infects leaves of both seedlings and adult plants. Individual lesions typically measure 4–10 mm in diameter; however, they frequently merge, resulting in extensive irregular necrotic areas, ultimately causing leaf desiccation and defoliation. Control of the disease can be difficult when the environment is conducive to disease development, especially in warm and humid climates (Blazquez 1967, Zhao *et al.* 2022). Moreover, in the future, the effects of climate change, especially increasing temperatures, may allow this disease to spread and become more severe even in regions where it has rarely occurred until now. In greenhouse cultivation, environmental controls such as the timing of the opening or closing of side curtains and vents are used to suppress the spread of this disease. Multiple fungicide applications have also been widely used to prevent the outbreak and spread of the disease. However, these methods have costs associated with time, labor, and materials: they require training and experience in determining the optimal frequency and timing of fungicide sprays, as well as in the effective use of environmental controls. In addition, the emergence of TLS isolates resistant to fungicides is a contributing factor to the recent surge in disease incidence in cucumber (Miyamoto *et al.* 2009, Sun *et al.* 2022). Genetically conferred host resistance represents the most effective strategy for addressing current and future challenges in the global management of TLS. Consequently, the utilization of new cultivars with high-level resistance is vital.

The first report of inheritance of TLS resistance in cucumber involved resistance conferred by a single dominant gene, *Cca*, in cultivar ‘Royal Sluis 72502’ (Abul-Hayja *et al.* 1978). Marker CSFR33 was found to be linked to the recessive susceptibility allele, *cca-1*, carried within a Chinese germplasm source (‘Q5’) that was resistant to TLS (Fu *et al.* 2012, Wang *et al.* 2010). Genetic analysis of a resistant accession, PI 183976 (wild cucumber *C. sativus* var. *hardwickii*), revealed that its resistance was controlled by a single recessive gene, *cca-2* (Wen *et al.* 2015, Xu *et al.* 2025). A recessive resistance locus, *cca-3*, in inbred line D31 (originated from resistant accession WI2757) was also fine-mapped to Chr. 6, and a candidate gene (Csa6M375730) at this locus, encoding a CC (coiled-coil motif)–NB–ARC (central nucleotide-binding domain) type R-gene analog, is considered to be responsible for the hypersensitive response to infection by the TLS pathogen in cucumber (Wen *et al.* 2015). More recently, three loci (*gTLS5.1*, *gTLS5.2*, and *gTLS7.1*) associated with TLS were identified by a genome-wide association study (GWAS), and one to three candidate genes were suggested by gene expression and haplotype analysis (Xu *et al.* 2025). The molecular mechanisms underlying the interaction between cucumber and *C. cassiicola* are gradually becoming clearer. The two Mildew Resistance Locus O genes, *CsMLO1* and *CsMLO2*, were reported to be negative regulators of cucumber defense response to TLS (Yu *et al.* 2019). Two other cucumber genes, a sucrose non-fermenting 1-related protein kinase (*CsSnRK1*) gene (Huang *et al.* 2023) and a caffeoyl shikimate esterase (*CsCSE5*) gene (Yu *et al.* 2023), positively modulate cucumber growth and resistance to TLS. In addition, plant Rho (ROP) proteins such as *CsROP5* and *CsROP10* are thought to be involved in TLS resistance by regulating Rho-related guanosine triphosphatases (ROP GTPases) and ABA signaling pathways (Yu *et al.* 2024).

Japanese cucumber constitutes a genetically distinct subpopulation that has undergone long-term regional selection, resulting in unique allelic compositions and phenotypic characteristics compared with Chinese, South Asian, and European cucumber groups (Shigita *et al.* 2024). Because disease resistance phenotypes often arise from population-specific adaptation, Japanese cucumber may harbor resistance loci and regulatory mechanisms that may differ substantially from those described in other cucumber types. In fact, commercial cultivars resistant to TLS have been successfully developed, and TLS damage has decreased compared to the past, at least in Japan. The TLS-resistant cultivars, combined with other favorable agronomic traits, reduce the risk of yield loss caused by this disease and are valuable options for growers. However, it is common for plant resistance to be overcome by pathogens. For example, a single recessive resistance locus, *dm1*, was effective in controlling downy mildew (DM; caused by *Pseudoperonospora cubensis*) and has been extensively used in cucumber breeding for nearly 60 years, but this locus has no longer been fully effective since the emergence of new high-virulence DM strains in the USA (Holmes *et al.* 2004, Thomas *et al.* 2017). In addition, this locus is also known to be involved in resistance to anthracnose (caused by *Colletotrichum* *orbiculare*) (Wang *et al.* 2019), but it is no longer effective for some strains of anthracnose (Matsuo *et al.* 2022). Therefore, it is important to identify new resistance genes (loci) that may help overcome future epidemics driven by the emergence of new TLS races. Furthermore, the development of molecular breeding techniques related to new resistance genes (loci) would be useful for the rapid development of new resistant cultivars.

As mentioned earlier, several studies have partially elucidated the genetic mechanisms underlying TLS resistance; however, the mechanisms responsible for TLS resistance in Japanese-type cucumber remain poorly understood. In this study, we aimed to clarify the genetic mechanisms of TLS resistance in a Japanese cucumber line for which the genetic factors involved in resistance are unknown. To do this, we constructed a genetic map using F_2_ populations derived from a cross between resistant and susceptible lines by restriction-site-associated DNA sequencing (ddRAD-seq) analysis, and we performed quantitative trait locus (QTL) analysis for resistance to *C. cassiicola* based on phenotyping of F_2:3_ families. The effects of major QTLs and QTL combinations were re-evaluated using selected F_2:3_ families, and single-nucleotide polymorphism (SNP) markers linked to each QTL were tested using segregating populations derived from crosses between the resistant line and other susceptible lines to evaluate the effectiveness of marker-assisted selection in breeding. In addition, a major QTL region was narrowed down using recombinant populations, and candidate genes within the region were identified.

## Materials and Methods

### Plant and fungal materials

A Japanese-type cucumber inbred line, TCS-J1, and a Beit Alpha–type inbred line, TCS-B1, both preserved at the University of Tsukuba, Japan, were used as resistant and susceptible parental lines, respectively, for QTL analysis. They were crossed to obtain the reciprocal F_1_s, F_1_A (TCS-B1 [♀] × TCS-J1 [♂]) and F_1_B (TCS-J1 [♀] × TCS-B1 [♂]), and their F_2_ and F_2:3_ families were obtained by self-pollination of one F_1_A individual and 183 F_2_ individuals, respectively. F_4:5_ and F_5:6_ populations were developed from the selected F_2_ and F_2:3_ individuals for fine mapping.

Three additional F_2_ populations were developed from crosses between TCS-J1 and three inbred lines with different genetic backgrounds preserved at the University of Tsukuba, Japan: a slicing-type cultivar, designated PO (PO [♀] × TCS-J1 [♂]); an old Japanese-type cultivar, designated TO (TO [♀] × TCS-J1 [♂]); and an old Chinese-type cultivar, designated G100 (TCS-J1 [♀] × G100 [♂]). Approximately 160 individuals of each F_2_ population were used for QTL validation.

All inoculations were performed with *C. cassiicola* strain Tcc-01, which was provided by the Institute of Vegetable and Floriculture Science, National Agriculture and Food Research Organization in Japan. The strain was propagated on potato dextrose agar (PDA; Nissui Pharmaceutical Co., Ltd., Tokyo, Japan) and incubated at 25°C for 7 days to 12 days in the dark before the inoculation tests.

### Evaluation of resistance to TLS

Evaluation of resistance to TLS for QTL analysis was performed using three different assays: a detached-cotyledon assay, conducted in 2018; a cotyledon assay, conducted in 2020 and 2023; and a seedling assay, conducted in 2020. In all assays for QTL analysis, both parent lines and reciprocal F_1_s were tested as controls in addition to the 183 F_2:3_ families.

In the detached-cotyledon assay, 28 plants of each F_2:3_ family were grown in 60-cell trays for 12 days after sowing in plant growth chambers maintained at 25°C under a 12-h photoperiod. Fully expanded cotyledons and the hypocotyl were cut together from each plant and placed in cell-plastic boxes filled with a small amount of water so that the cut edge of the hypocotyls was soaked in water. Then, each cotyledon was inoculated with a 20-μL drop of spore suspension at 8 × 10^4^ sporangia mL^–1^ using a digital micropipette. The inoculated cotyledons were incubated at 25°C with nearly 100% humidity in the dark for 36 h, and then placed under a 12-h photoperiod for 7 days at 25°C. Lesions on cotyledons were photographed with a digital camera (EOS Kiss X9, Canon Inc., Tokyo, Japan). The lesion area as a percentage of the whole cotyledon area (PLA) was calculated by ImageJ software (Schneider *et al.* 2012). The PLA average of 24 to 28 plants per F_2:3_ family was used for QTL analysis.

In the cotyledon assay, 30 plants of each F_2:3_ family in 2020 and 28 plants in 2023 were grown in 60-cell trays for 10 days after sowing in plant growth chambers maintained at 25°C under a 12-h photoperiod. Then, conidial suspensions with 2.5 × 10^5^ sporangia mL^–1^ in 2020 and 3.0 × 10^5^ sporangia mL^–1^ in 2023 were sprayed evenly on both expanded cotyledons. In both these and the seedling assays, 0.5 μL/mL Tween-20 (Kanto Chemical Co., Inc., Tokyo, Japan) was added to the inoculum. In the seedling assay, eight seeds of each F_2:3_ family were sown in 7.5-cm-diameter pots filled with soil and placed in a plant growth chamber maintained at 28°C under a 12-h photoperiod for 2 days, followed by 25°C under a 12-h photoperiod for 4 days, followed by 25°C during the day and 18°C at night for 11 days. A conidial suspension with 2.5 × 10^5^ sporangia mL^–1^ was then sprayed on the surface of the first true leaf. The inoculated plants were incubated at 25°C in the dark with nearly 100% humidity for 36 hours and then moved out at 25°C under a 12-h photoperiod. Disease severity (DS) was evaluated visually according to lesion size and number at 9 days after inoculation (DAI) in the cotyledon assay and 11 DAI in the seedling assay on the following scale (Supplemental Table 1): 0, no lesions; 1, a few small lesions; 2, small lesions or a few medium lesions; 3, medium lesions, or many small lesions and a few medium lesions, or a few large lesions; 4, large part of cotyledon or true leaf is withered; 5, more than half of cotyledon or true leaf is withered; and 6, cotyledon or true leaf is completely dead. The average DS score of plants within each F_2:3_ family in each assay was used for QTL analysis.

### ddRAD-seq analysis and linkage map construction

Genomic DNA was extracted from parental lines and 183 F_2_ individuals using the DNeasy Plant Kit 96 (Qiagen, Hilden, Germany). Library construction and sequencing analysis were performed as described by Shirasawa *et al.* (2016) with minor modifications. Two restriction enzymes, *Pst*I and *Msp*I (Thermo Fisher Scientific Inc., Waltham, MA, USA) were used to construct the ddRAD-Seq libraries. Digested DNA was ligated to adapters using T4 DNA ligase (Takara Bio Inc., Kusatsu, Japan) and purified with Agencourt AMPure XP reagent (Beckman Coulter, Brea, CA, USA) to remove short fragments (<300 bp). The purified DNA was PCR-amplified with indexed primers, and PCR products were purified using the QIAquick PCR Purification Kit (Qiagen). Fragments (300–1000 bp) were separated on a 2.0% agarose gel (Kanto Chemical Co., Inc.) and extracted with a MinElute Gel Extraction Kit (Qiagen). The libraries were sequenced on a HiSeq 4000 (Illumina Inc., San Diego, CA, USA) in 100-bp paired-end reads. The data were mapped to the *C. sativus* reference genome, Chinese Long v2 (Huang *et al.* 2009), in Bowtie 2 (Langmead and Salzberg 2012). SNPs calling was performed by BCFtools v.1.19 (Li *et al.* 2009).

A genetic linkage map was constructed based on the SNPs in R/ASMap (Taylor and Butler 2017) and R/qtl (Arends *et al.* 2010). SNP markers were selected by excluding loci where more than 20% of progeny had missing values. F_2_ individuals that could be genotyped with >80% of all SNP markers were selected. Then, *p*-values were calculated to test for deviations of genotype frequencies at each locus from the expected ratio of 1:2:1, and after Bonferroni correction for multiple testing, markers that showed significant distortion at the 5% level were removed. Genotype error rate and genotype-error logarithm of the odds (LOD) scores were calculated, and SNP genotypes with high genotype-error LOD score (>0) were treated as missing data. Genetic distance, the order of SNP markers, and the imputation of missing genotypes were calculated using the function mstmap in R/ASMap and the minimum-spanning-tree (MST) algorithm. The Kosambi mapping function was applied during the calculation of the genetic map distance. The R package LinkageMapView (Ouellette *et al.* 2018) was used to visualize the linkage map. Composite interval mapping (CIM) was used to detect QTLs. The LOD significance was determined using 1000 permutations, and the 1.5-LOD support intervals for the location of the identified QTLs were calculated.

### Marker development and QTL validation

A SNP obtained by ddRAD-seq was chosen in each of the two major QTL regions on Chr.5 (*Tls5.1* and *Tls5.2*; see Results). These SNPs were used for selection of F_2:3_ families to confirm the effects of these QTLs by the seedling assay described above, with some modifications. Specifically, both cotyledons and true leaves were inoculated, and lesions on both organs were evaluated. The effects of these QTLs were further validated in four F_2_ populations derived from the cross of TCS-B1 × TCS-J1 and from crosses between TCS-J1 and three fixed lines with different genetic backgrounds (see the subsection “Plant and fungal materials”). Genotyping of F_2_ individuals was conducted by high-resolution melting (HRM) analysis using one marker for each QTL. HRM analysis was performed using QuantStudio^TM^ Real-time PCR system with MeltDoctor^TM^ HRM Master Mix (Thermo Fisher Scientific Inc.) in a 10-μL reaction according to the manufacturer’s recommendation. A melt curve analysis was performed with a temperature increment of 0.025°C/s from 60 to 95°C. The design of HRM primers was based on the Chinese Long genome v2 (Huang *et al.* 2009) and v3 (Li *et al.* 2019), using Primer3Plus (https://www.primer3plus.com). The resistances of approximately 160 individuals of each F_2_ population were evaluated by a modified seedling assay in which both cotyledons and true leaves were inoculated, and the DS score of both tissues was evaluated.

### Fine mapping of *Tls5.2*

The F_2_ individuals from TCS-B1 × TCS-J1 with recombination in *Tls5.2* were selected with 11 HRM markers and were self-pollinated to develop F_3_ families. Through repeated selection and selfing, nine F_4:5_ families and two F_5:6_ families were developed and used for fine mapping. The DS average of 8 to 16 plants in each family was obtained using a seedling assay as previously described, and the phenotype–genotype relationships were investigated. The QTL region was narrowed down, and genes were predicted on the basis on the Chinese Long genome v2 (Huang *et al.* 2009) and v3 (Li *et al.* 2019).

## Results

### Resistance to TLS in the mapping population

As expected, the resistant parent TCS-J1 was nearly completely resistant: it had the smallest PLA (0.15%) in the detached-cotyledon assay, average DS scores of 0.1 and 1.08 in two cotyledon assays (2020 and 2023), and an average DS score of 0.6 in the seedling assay (Fig. 1A). TCS-J1 had some small lesions, but the lesions stopped expanding and the majority of the cotyledons remained green at 9 DAI. In contrast, lesions of the susceptible parent TCS-B1 continued expanding, resulting in high PLA (50.9%) in the detached-cotyledon assay, high DS scores of 4.7 and 3.8 in the two cotyledon assays, and high DS of 4.0 in the seedling assay. On 9 DAI, the various-sized lesions on TCS-B1 leaves had merged frequently, forming extensive irregular necrotic areas that sometimes covered the entire leaf area. The DS of reciprocal F_1_s was intermediate between those of the two parental lines (Fig. 1A, 1B). No significant difference was observed between the reciprocal F_1_s in all assays. Their lesions had chlorotic boundaries, sometimes merged with other necrotic lesions nearby, but that expansion was limited. In the F_2:3_ families, PLA in the detached-cotyledon assay and DS scores in the cotyledon and seedling assays varied continuously (Fig. 1A).

### Linkage map construction and QTL analysis

In ddRAD-seq analysis of 183 F_2_ individuals, an average of 1.2 million reads per individual was obtained. After SNP calling and filtration, 1103 SNPs were used to construct the linkage map. The linkage map spanned 1009.7 cM, with an average distance between markers of 0.9 cM (Supplemental Fig. 1, Supplemental Table 1). For the QTL analyses, five F_2:3_ families were removed because of missing genotypes (>20%). QTL mapping in the remaining 178 F_2:3_ families using four assays detected a total of five QTLs on Chr. 2, Chr. 5, and Chr. 6 (Fig. 2, Supplemental Table 2). Among those, two QTLs on Chr. 5 were detected at least three assays, so they were named TLS-resistance QTLs (*Tls5.1* and *Tls5.2*) and further analyzed (Table 1). Focusing on the gene action of these two QTLs, the absolute values of the additive effect (*a*) were higher than those of the dominance effect (*d*) (Table 1). The |*d*/*a*| ratio for each QTL ranged from 0 to 0.2, suggesting that additive effects play an important role in this trait. The QTL genotype effects were indicated by plotting the average DS score for each genotype of the SNP closest to the LOD peak of each QTL in the mapping population (Supplemental Fig. 2). The phenotypic variance explained (PVE) by the QTLs ranged from 26.3% to 46.7% (*Tls5.1*), and from 29.7% to 48.5% (*Tls5.2*). No significant interaction (epistasis) between the two QTLs was detected by testing all possible pairwise interactions using the addint function in R/qtl.

### Marker development and confirmation of the effects of two major QTLs, *Tls5.1* and *Tls5.2*, on resistance

On the basis of the nearest SNP to the LOD peak of each major QTL, *Tls5.1* (SNP at Chr.5-17141939) and *Tls5.2* (Chr.5-24749906), 21 F_2:3_ families were selected: 7 homozygous for the resistance allele of both QTLs, 7 homozygous for the susceptibility allele of both QTLs, 5 homozygous for the resistance allele of *Tls5.1* and the susceptibility allele of *Tls5.2*, and 2 homozygous for the susceptibility allele of *Tls5.1* and the resistance allele of *Tls5.2* (Supplemental Table 3). As expected, F_2:3_ families homozygous for both resistance alleles had low DS scores, ranging from 0.9 to 1.9 for cotyledon and from 1.5 to 2.3 for true leaf (Fig. 3, Supplemental Table 3). In contrast, F_2:3_ families homozygous for both susceptibility alleles had high DS scores, ranging between 4.3 and 6.0 for cotyledon and from 2.7 to 4.3 for true leaf. F_2:3_ families homozygous for the resistance allele at either one of the two QTLs exhibited intermediate DS scores.

To verify the effect of the two QTLs in different genetic backgrounds, two developed HRM markers based on SNPs nearest to LOD peak at *Tls5.1* (Chr.5-16965758; named HRM-01) and *Tls5.2* (Chr.5-24646609; named HRM-04) (Supplemental Table 4), were selected for genotyping of individuals from four F_2_ populations, each produced by crossing TCS-J1 with a different susceptible parent. The marker genotypes were compared to the DS scores from cotyledon and true-leaf assays of the same individuals. For the cotyledon DS score, there were no significant differences among genotype groups in the F_2_ populations except for TCS-B1 × TCS-J1, although the parental lines significantly differed in each population (Fig. 4). However, there were significant differences between genotypes in the true-leaf DS score in all F_2_ populations. As expected, the genotype group homozygous for resistance alleles (derived from TCS-J1) at both loci showed lower true-leaf DS scores than other genotype groups, and the genotype group homozygous for susceptibility alleles at both loci showed the highest DS scores. Other genotype groups had DS scores of true leaves intermediate between those of these two groups (Fig. 4).

### Fine mapping of *Tls5.2*

Among the QTLs detected in the different assays, *Tls5.2*, which showed a larger effect than *Tls5.1* in the seedling assay, was targeted for fine mapping. Eleven polymorphic HRM markers were designed to be as equally spaced as possible within the 1.5-LOD support interval region of *Tls5.2* (Supplemental Table 4). By using markers on both ends of the *Tls5.2* region, 32 F_2_ individuals with recombination in this region were identified; the relationship between the marker genotype of each F_2_ individual and the DS score of its F_2:3_ family indicated that the causal gene is located between HRM-02 and HRM-09 (Fig. 5). Subsequently, seven F_4:5_ families and one F_5:6_ family were developed and used for further fine mapping. The relationship between marker genotype and DS score in these generations narrowed down the QTL region to between HRM-06 (Chr.5-24670681) and HRM-08 (Chr.5-25167471) (Fig. 5). Within this 496.8-kb region, 69 genes are annotated in the Chinese Long v2 and v3 reference genomes, including several candidates potentially involved in plant disease resistance, such as genes encoding protein kinase-like domains and members of the GATA transcription factor family (Supplemental Table 5).

## Discussion

So far, TLS-resistance genes or loci have been identified on six chromosomes (all except for Chr. 4) in past studies using different resistance sources. In this study, we revealed the polygenic control of resistance in the Japanese fixed line TCS-J1 through the detection of five loci on three chromosomes (Chr. 2, Chr. 5, and Chr. 6; Table 1, Supplemental Table 2). All five QTLs were located far from previously identified resistance loci. For example, past studies reported three resistance loci on Chr. 6, namely *cca-1*, *cca-2*, and *cca-3* (Wen *et al.* 2015, Xu *et al.* 2025), but the location of the QTL on Chr. 6 detected in this study was not near any of them. The three QTLs detected on Chr. 5 were also located far from previously identified resistance loci on that chromosome, *gTLS5.1* and *gTLS5.2* (Xu *et al.* 2025) and *CsCSE5* (Yu *et al.* 2023). Although the sample size for each phenotypic evaluation in our QTL analysis was relatively limited, we conducted four independent assays to ensure robustness. The QTLs that were consistently detected, particularly *Tls5.1* and *Tls5.2*, are highly likely to contribute to TLS resistance in TCS-J1. Since these QTLs were first identified in this study, the origin of resistance in TCS-J1, a fixed line derived from a modern Japanese cultivar, is likely to differ from that of the resistant materials used in previous research.

Among the QTLs detected in this study, two QTLs on Chr. 5 (*Tls5.1* and *Tls5.2*) were detected in three different types of assays. These QTLs had additive effects on DS score in both cotyledon and true-leaf assays (Supplemental Fig. 2). Since the effects of these QTLs were confirmed in additional seedling assays using F_2:3_ families (Fig. 3, Supplemental Table 3), we conclude that they contribute significantly to the resistance of TCS-J1 to TLS. Considering PVE in the QTL analyses (Table 1), both loci had roughly equivalent effects in cotyledons, and *Tls5.2* had a higher effect in true leaves. However, there was no significant difference in the DS score on true leaves between the two F_2:3_ families homozygous for resistance at one locus and susceptibility at the other (Fig. 3), indicating that the two QTLs may have roughly the same effect even on TLS resistance of true leaves. In any case, homozygosity at both loci is required to confer high resistance close to that of TCS-J1. Furthermore, because the effects of these QTLs were validated in the progeny of crosses with lines (or cultivars) of different genetic backgrounds, we expect them to be effective in a variety of cucumber types.

Strong relationships between resistance to different pathogens have often been observed in cucumber breeding, and previous mapping studies have revealed the co-localization of many disease resistance QTLs (reviewed in Wang *et al.* 2020). In particular, Chr. 5 and Chr. 6 contain several hotspots that are highly enriched in resistance genes against various pathogens. Two QTLs detected in this study were also located near several resistance QTLs for other diseases. *Tls5.1* is close to *DM5_2*, *dm5.2*, *gsb5.1*, and *pm5.*2, while *Tls5.2* is overlaped *pm5.3* and *dm5* (Shimomura *et al.* 2021, Wang *et al.* 2020, Zhang *et al.* 2018). It is unclear what alleles for disease resistance loci other than *Tls5.1* and *Tls5.2* are carried on Chr. 5 of TCS-J1, but if any of these are negative alleles, introducing *Tls5.1* and *Tls5.2* from TCS-J1 may affect the resistance level of other diseases owing to linkage drag. Thus, the development of DNA markers tightly linked to the two TLS-resistance loci is vital for breeding lines with resistance to multiple diseases. In this study, we developed one HRM marker for *Tls5.1* and 11 HRM markers for *Tls5.2* (Supplemental Table 4). The former marker, named HRM-01 (Supplemental Table 4), is located 176,181 bp away from the LOD peak, and its genotype appears to be associated with resistance level (Figs. 3, 4). The *Tls5.2* region was successfully narrowed down to 496.8 kb by fine mapping, and five markers in this region, HRM-04 to HRM-08, also appear to be linked strongly to the causal gene(s) for this QTL (Figs. 3, 5). Therefore, these HRM markers, while still requiring further validation, are useful for MAS to develop TLS-resistant cultivars.

*Tls5.2* was narrowed down to a 496.8-kb region with 69 candidate genes (Supplemental Table 5). In the cucumber genome, approximately 178–192 genes encode receptor-like kinases (RLKs), 42–56 encoding receptor-like proteins (RLPs) (Berg *et al.* 2020, Zhang *et al.* 2021a), and 67–70 encoding immune receptors (NLRs) (Morata and Puigdomènech 2017, Wan *et al.* 2013, Yang *et al.* 2013). Some of the proteins in these categories confer specific resistance to several diseases. Among the 69 genes in the *Tls5.2* region, two genes, Csa5G623450 and Csa5G623910, that encode a protein kinase-like domain (as do many R genes), may be involved in TLS resistance of TCS-J1. Csa5G623920.1, a *Sigma factor binding protein 1* gene, acts as an R gene mediating host defense response for cucumber downy mildew resistance (Tan *et al.* 2022). Csa5G622830, which belongs to the GATA gene family, was reported to be simultaneously active in resistance to downy mildew, powdery mildew, and root-knot nematode (Zhang *et al.* 2021b). Previous research has shown that there are common genes, such as the MLO gene family (Berg *et al.* 2015, Yu *et al.* 2019) and the CSE gene family (Yu *et al.* 2023), that confer resistance to TLS and other diseases. Because of their potential roles in disease resistance, they are considered potential candidates for the gene(s) underlying *Tls5.2*; however, at this stage, the possibility that other genes within this region may be responsible cannot be ruled out. In the future, it will be necessary to conduct a detailed analysis of the candidate genes within the *Tls5.2* region, which includes these four genes, and to subsequently verify their functions and roles in TLS resistance through comprehensive expression profiling and knockout or knock-in approaches using genetic transformation or genome editing techniques.

This study demonstrated that TLS resistance in an inbred line derived from a modern Japanese cultivar is controlled by multiple loci distinct from those previously reported. The resistance conferred by the two major QTLs, Tls5.1 and Tls5.2, which have roughly equivalent effects, is high—although not to the same level as that of TCS J1—and both exhibit strong additive effects. Disease resistance is generally a highly complex trait involving multiple genes and mechanisms, and the QTLs identified in this study represent only part of the overall resistance architecture. Future research should aim to clarify the complete picture of TLS resistance in cucumber, including potential interactions between the QTLs detected here and other known resistance genes or loci. Although some challenges remain, our findings provide valuable genetic insights for a better understanding of TLS resistance in cucumber. These newly identified loci may contribute to more robust resistance when combined with previously identified resistance genes or loci. Furthermore, our results highlight the potential of using molecular markers to select for TLS resistance derived from TCS-J1, thereby enabling more efficient breeding strategies against this disease.

## Author Contribution Statement

YY conceived and supervised the study. YY and TMH developed the populations. TMH and RC evaluated phenotypes. SI and KS performed ddRAD sequencing. TMH and RC built the linkage map and performed QTL analysis. TMH performed fine mapping and marker analysis. TMH and YY wrote, reviewed, and edited the manuscript. All authors reviewed the manuscript.

## Supplementary Material

Supplemental Figures

Supplemental Tables

## Figures and Tables

**Fig. 1.**
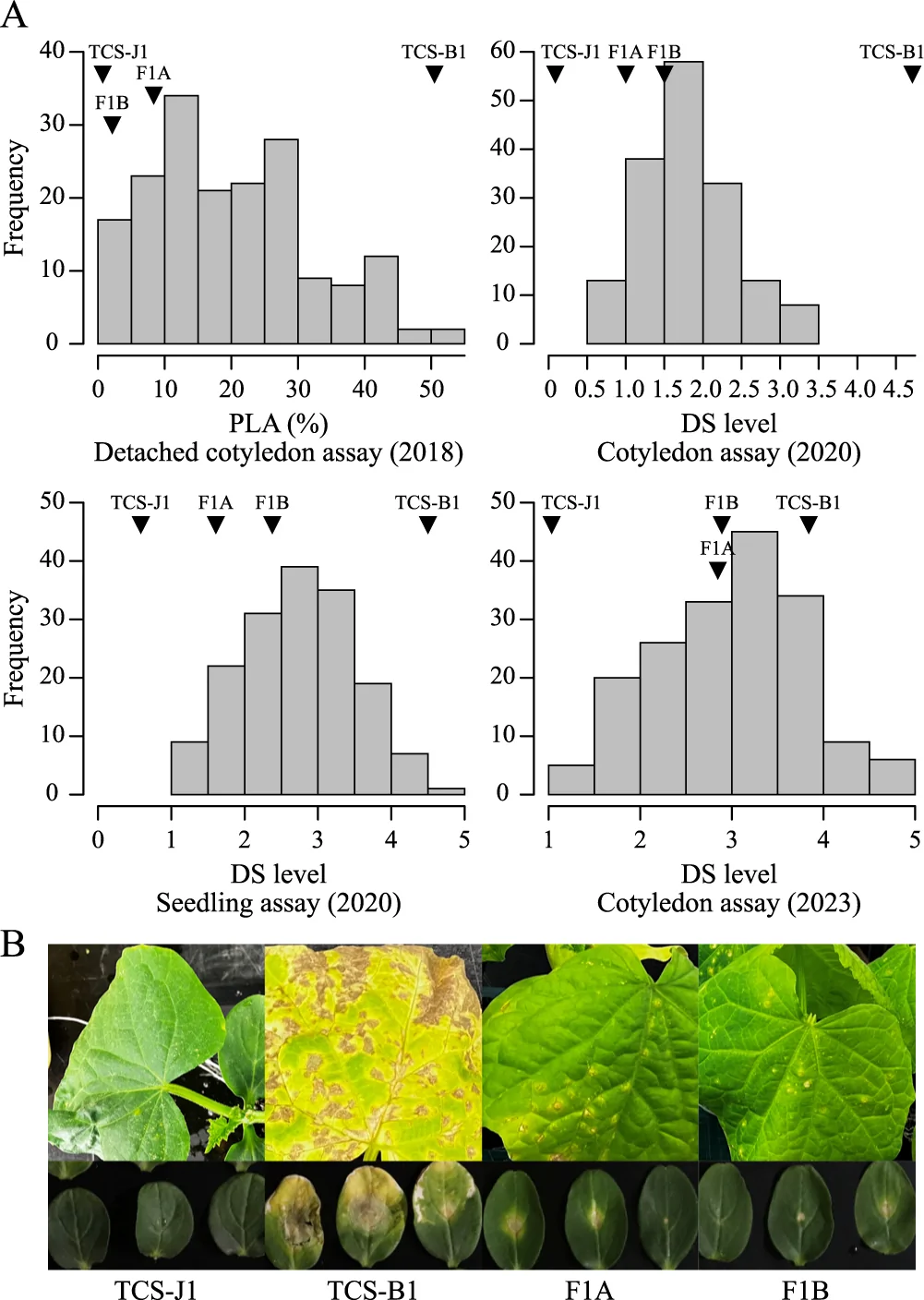
Disease severity in parents and progeny of crosses between TCS-B1 and TCS-J1. (A) Bars indicate frequency distribution of disease severity levels in 183 F_2:3_ families derived from TCS-B1 (♀) × TCS-J1 (♂) by four different assays. Arrows indicate means of the parents and reciprocal F_1_s between TCS-B1 and TCS-J1. DS, disease severity score; PLA, lesion area as a percentage of the whole cotyledon area. (B) Typical symptoms of parental lines and their reciprocal F_1_s in the seedling assay (top) and detached-cotyledon assay (bottom). F1A, TCS-B1 (♀) × TCS-J1 (♂); F1B, TCS-J1 (♀) × TCS-B1 (♂).

**Fig. 2.**
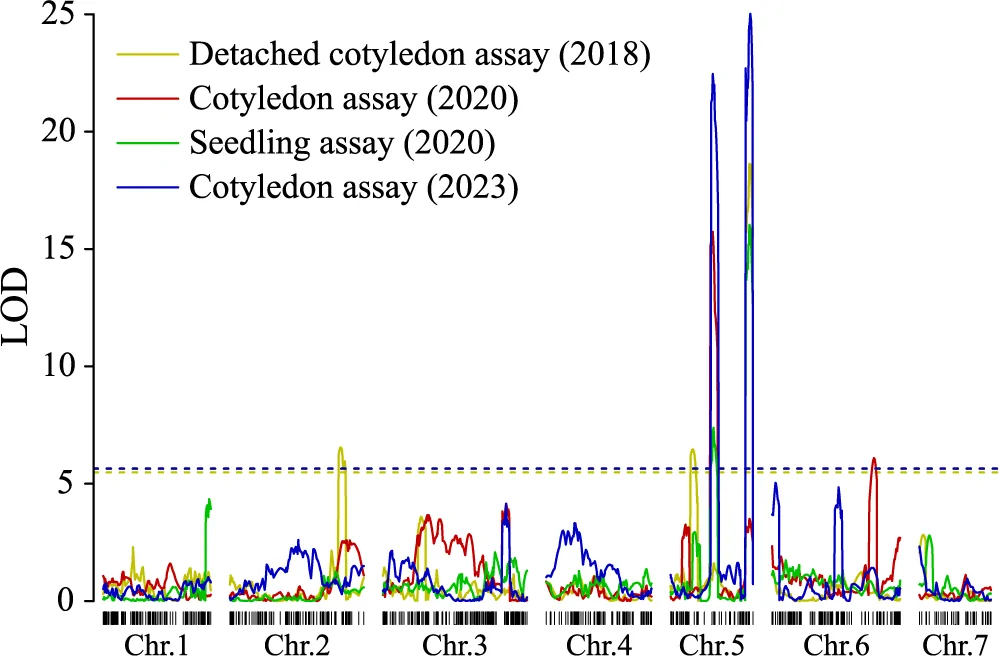
Logarithm of odds (LOD) scores associated with target leaf spot disease severity measured in four different assays. The horizontal dashed line indicates the LOD threshold.

**Fig. 3.**
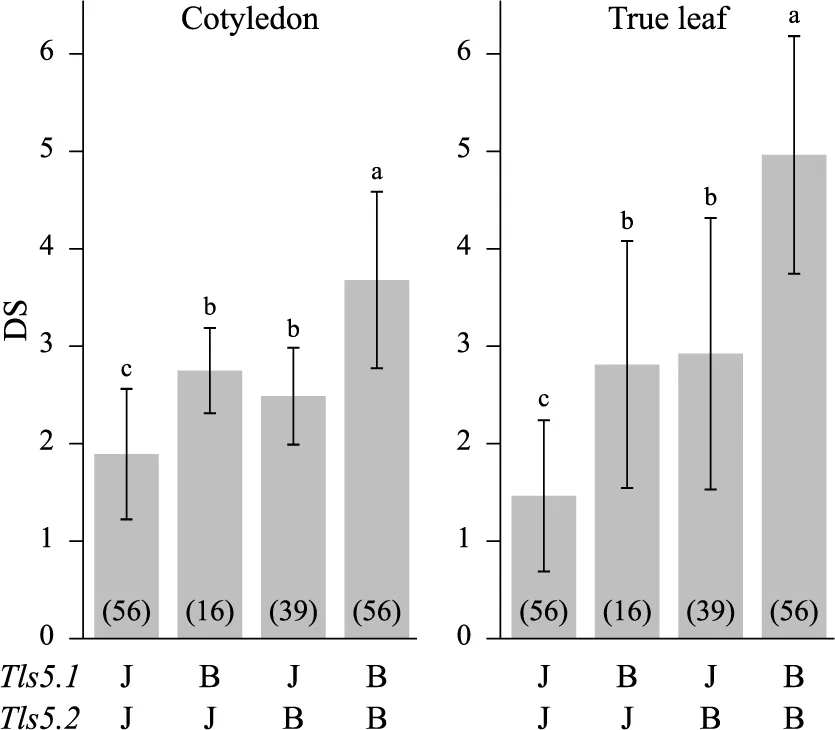
Effect of two QTLs, *Tls5.1* and *Tls5.2*, on disease severity (DS) scores of cotyledon and true leaf in selected F_2:3_ families derived from TCS-B1 (♀) × TCS-J1 (♂). The individuals from 21 F_2:3_ families were pooled into four genotype groups determined based on homozygosity of alleles from TCS-J1 (J alleles) and TCS-B1 (B alleles) at markers HRM-01 and HRM-04. Numbers in parentheses indicate the number of plants. DS scores marked with the same letter are not significantly different (*p* < 0.05) according to the Tukey–Kramer HSD test.

**Fig. 4.**
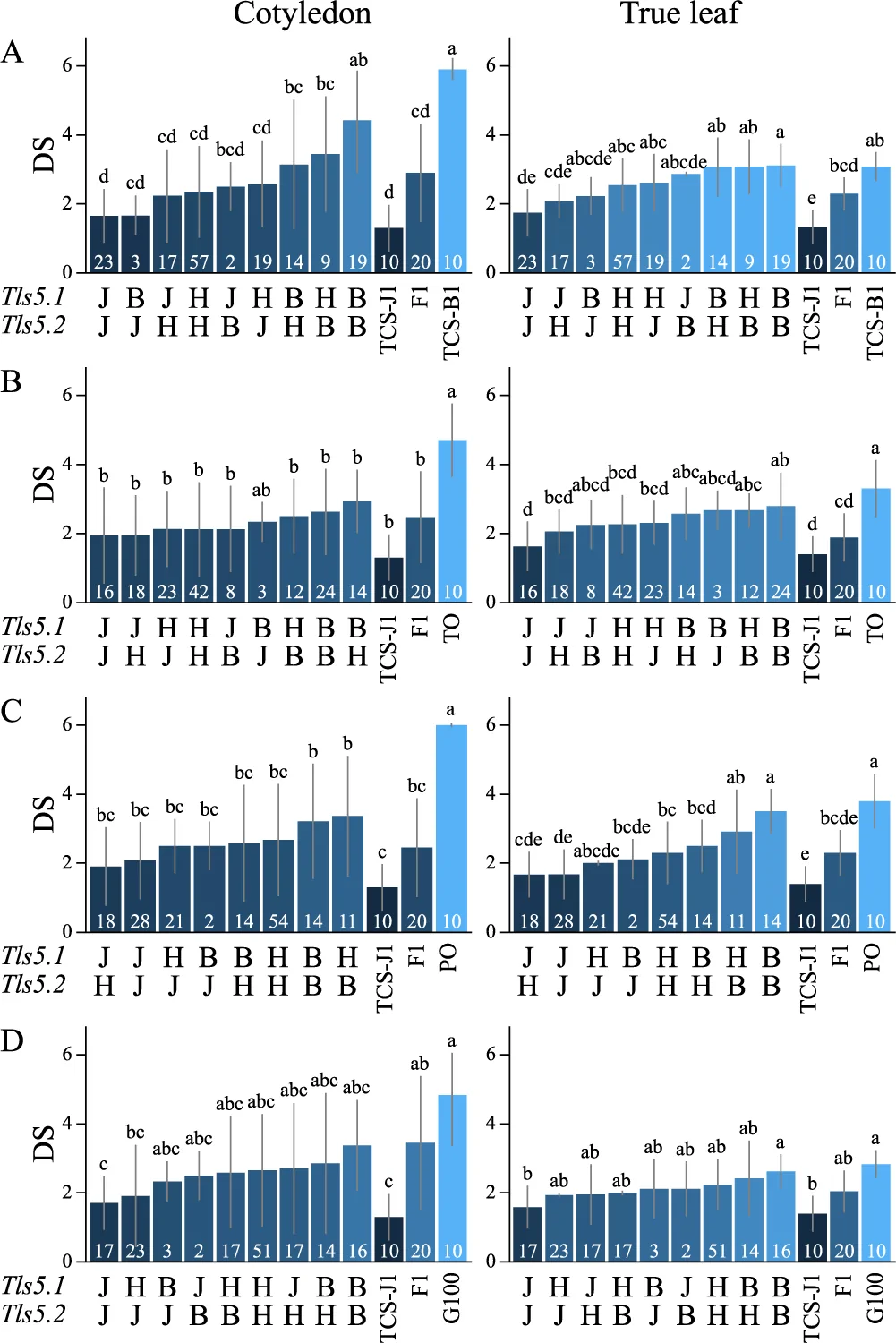
Validation of two QTLs, *Tls5.1* and *Tls5.2*, using four F_2_ populations derived from crosses between the resistant parent TCS-J1 and four fixed lines with different genetic backgrounds. Crosses were between TCS-J1 and (A) a Beit Alpha–type fixed line (TCS-B1), (B) a slicing-type cultivar (PO), an (C) an old Japanese-type cultivar (TO), and (D) an old Chinese-type cultivar (G100). Disease severity (DS) scores marked with the same letter are not significantly different (*p* < 0.05) according to the Tukey–Kramer HSD test. B, susceptible; J, resistant; BJ, heterozygous.

**Fig. 5.**
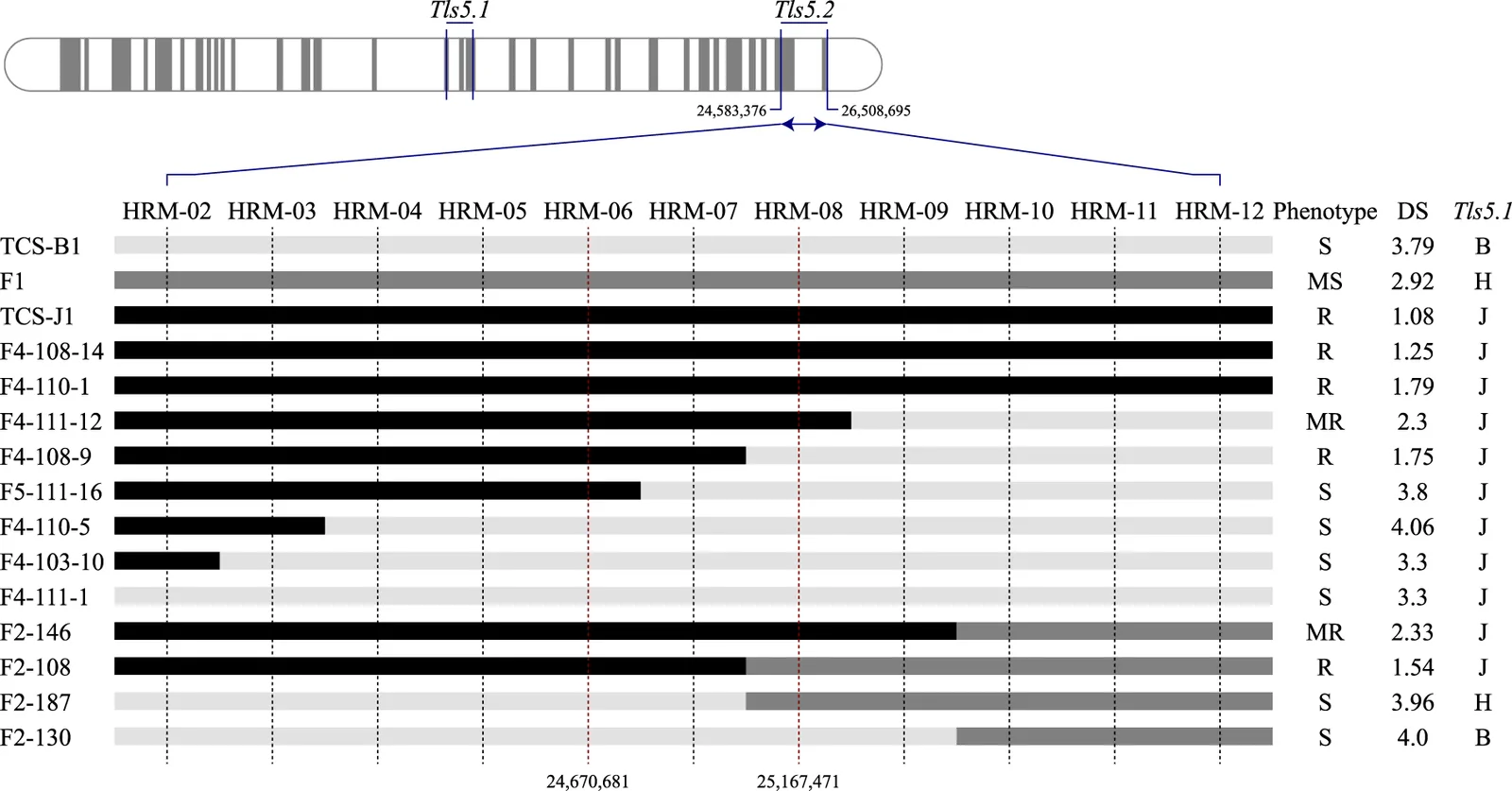
Fine mapping of *Tls5.2*. Fine mapping with F_2:3_, F_4:5_, and F_5:6_ individuals derived from TCS-B1 × TCS-J1 narrowed down the *Tls5.2* region to 496.8 kb (delineated by vertical red lines) with 69 genes reported in the Chinese Long v2 and v3 reference genome. Black, gray, and white bars indicate genotypes homozygous for the resistance (TCS-J1) allele, heterozygous, and homozygous for the susceptibility (TCS-B1) allele, respectively DS, disease severity score; B, susceptible; J, resistant; BJ, heterozygous.

**Table 1.** QTLs for target leaf spot resistance detected in at least three assays

QTL*^a^*	Assays	Chr.	Position (cM)	LOD peak (cM)	PVE (%)*^b^*	Additive effect (*a*)*^c^*	Dominance effect (*d*)	|*d*/*a*| ratio	Range (cM)*^d^*	Range (Mb)*^e^*
*Tls5.2*	Detached cotyledon	5	103	18.49	29.7	–9.47	–0.50	0.05	101.5–107.5	24.58–26.51
	Cotyledon (2023)	5	104	25.00	48.5	–0.80	0.06	0.08	101.5–107.5	24.58–26.51
	Seedling assay	5	104	15.86	42.6	–0.69	–0.04	0.06	100.8–107.5	24.66–26.50
*Tls5.1*	Cotyledon (2020)	5	55	15.73	30.9	–0.49	0.11	0.23	43.9–57.1	13.73–17.32
	Cotyledon (2023)	5	55	22.45	46.7	–0.80	0.13	0.16	52.0–60.0	16.13–17.37
	Seedling assay	5	55	5.84	26.3	–0.57	0.03	0.06	52.0–60.0	16.13–17.37

*^a^* QTL names were assigned according to the current nomenclature recommendations (Wang *et al.* 2020). *^b^* Percent of the phenotypic variation explained by the QTL. *^c^* Additive effect is the effect of substituting a TCS-J1 (J) allele for a TCS-B1 (B) allele; its negative value indicates that TCS-J1 has the negative allele. *^d^* Genetic distance of the 1.5-LOD interval of the QTL. *^e^* Physical distance of the 1.5-LOD interval in the Chinese Long v2 reference genome.
