# Supplementary material for: QTL mapping for target leaf spot resistance in a Japanese-type cucumber
Source: Breed Sci. 2026 Jul 2;76(3):309–17. doi: 10.1270/jsbbs.26005 (PMC13709615; doi:10.1270/jsbbs.26005)
Supplement: Supplementary file 1 [file 76_309_s1.pdf]

Ho et al./Breeding Science
doi: 10.1270/jsbbs.26005

Supplemental Fig. 1.

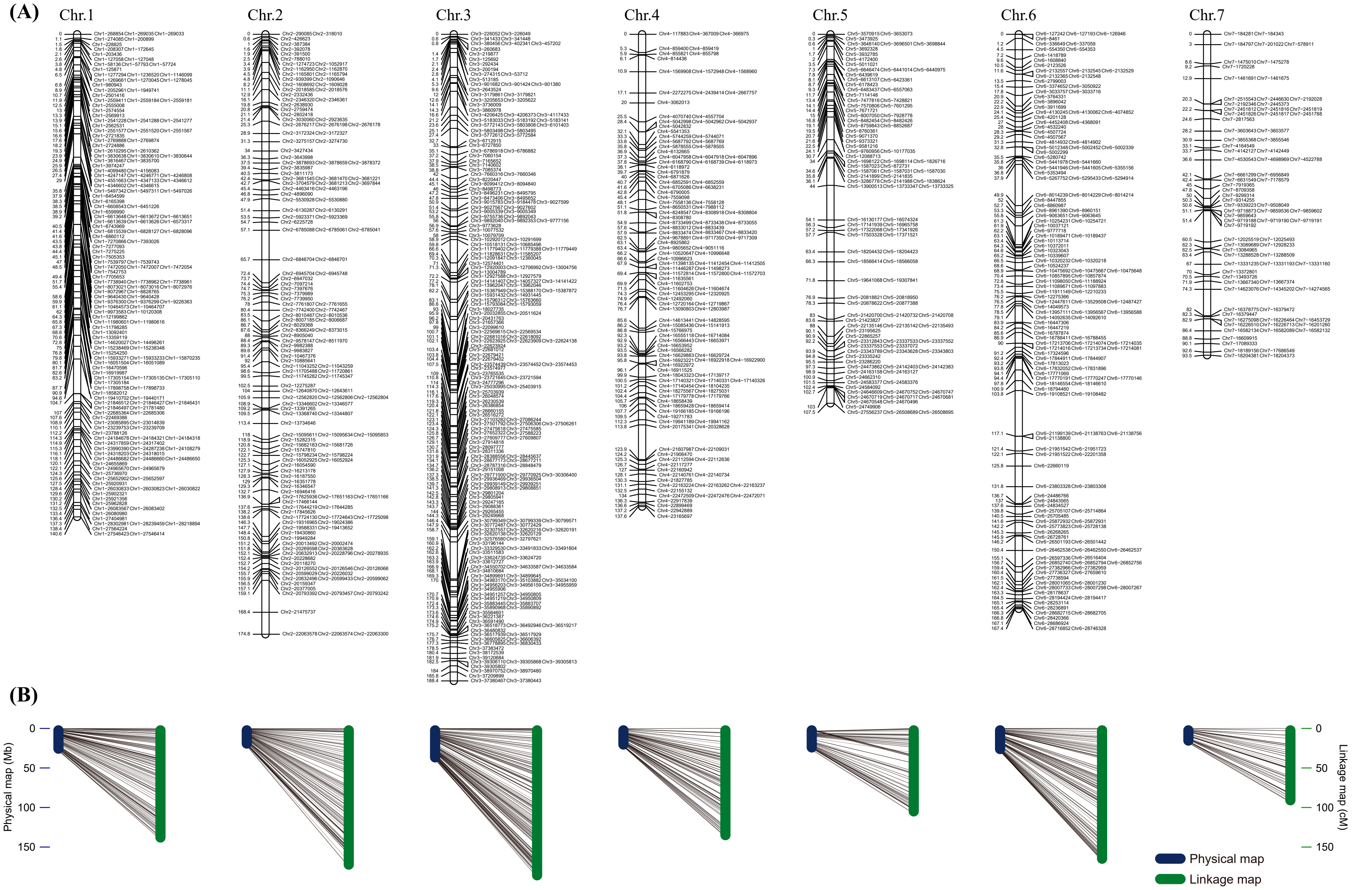

Supplemental Fig. 1. Linkage map constructed using the F_2 population derived from TCS-B1 (♀) × TCS-J1 (♂).
(A) Linkage map. (B) Collinearity between physical and linkage maps

Supplemental Fig. 2.

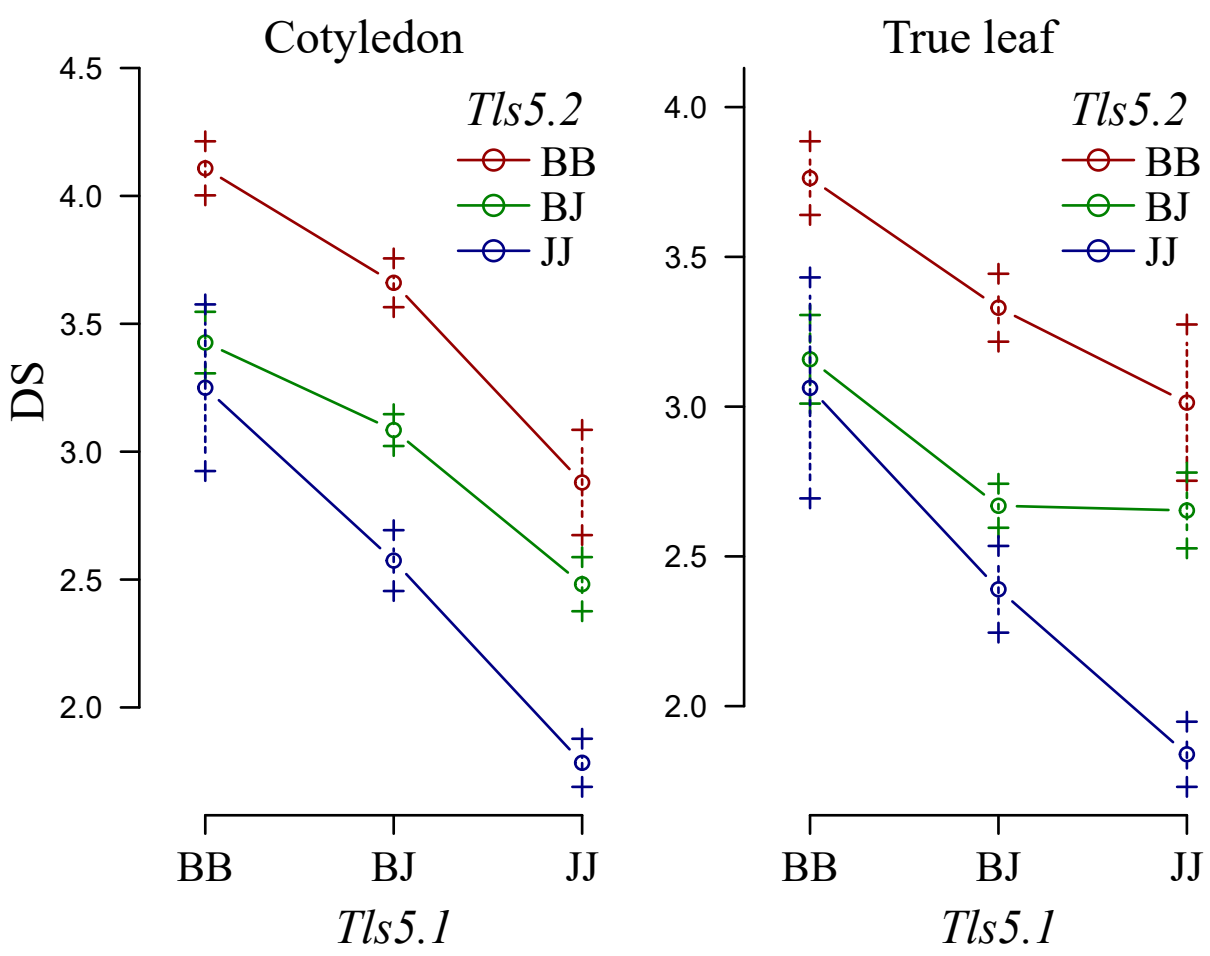

Supplemental Fig. 2. Effect of two major QTLs, *Tls5.1* and *Tls5.2*, on the disease severity (DS) score in the cotyledon assay conducted in 2023 and the seedling assay. BB: homozygotes for the susceptible (TCS-B1) allele, BJ: heterozygotes, JJ: homozygotes for the resistance (TCS-J1) allele
