# Supplementary material for: QTL mapping for target leaf spot resistance in a Japanese-type cucumber
Source: Breed Sci. 2026 Jul 2;76(3):309–17. doi: 10.1270/jsbbs.26005 (PMC13709615; doi:10.1270/jsbbs.26005)
Supplement: Supplementary file 2 [file 76_309_s2.pdf]

doi: 10.1270/jsbbs.26005

Supplemental Table 1. Distribution of SNP markers on 7 linkage groups

Chr.	Number of SNPs	Total length (cM)	Average distance (cM)	Max distance (cM)
1	184	140.6	0.8	10.1
2	164	174.8	1.1	9.3
3	238	188.4	0.8	10.8
4	148	137.6	0.9	10.1
5	112	107.5	1	10.1
6	172	167.4	1	13.3
7	85	93.5	1.1	9.1

Supplemental Table 2. QTLs for target leaf spot resistance detected in 183 $F_{2:3}$ familes derived from TCS-B1 (♀) × TCS-J1 (♂)

Assays	Chr.	Possition (cM)	LOD peak (cM)	PVE (%)[a]	Additive effect (*a*)	Dominance effect (*d*)	|*d*/*a*| ratio	Range (cM)[b]	Range (Mb)[c]
Detached cotyledon	2	145	6.26	6.6	3.78	3.75	0.99	138.6–151.2	17.77–20.01
	5	29	6.42	13.5	-6.47	-3.13	0.48	26.0–35.0	10.17–21.41
	5	103	18.49	29.7	-9.47	-0.50	0.05	101.5–107.5	24.58–26.51
Cotyledon (2020)	5	55	15.73	30.9	-0.49	0.11	0.23	43.9–57.1	13.73–17.32
	6	133	6.06	12.8	-0.30	0.02	0.07	125.8–137.0	22.66–24.48
Cotyledon (2023)	5	55	22.45	46.7	-0.80	0.13	0.16	52.0–60.0	16.13–17.37
	5	104	25.00	48.5	-0.80	0.06	0.08	101.5–107.5	24.58–26.51
Seedling	5	55	5.84	26.3	-0.57	0.03	0.06	52.0–60.0	16.13–17.37
	5	104	15.86	42.6	-0.69	-0.04	0.06	100.8–107.5	24.66–26.50

[a]Percent of the phenotypic variation explained by the QTL

[b]Genetic distance of the 1.5 LOD interval of the QTL

[c]Physical distance of the 1.5 LOD interval in Chinese long v2 reference genome

Supplemental Table 3. Average disease severity (DS) scores of each of 21 $F_{2:3}$ families derived from TCS-B1 (♀) × TCS-J1 (♂), used for confirmation of QTL effects by seedling assay

$F_{2:3}$ family ID	Number of plants	*Tls5.1* [a]	*Tls5.2* [a]	Average DS of cotyledon	Average DS of true leaf
CCR09_F2-030	8	B	B	4.4	3.6
CCR09_F2-033	8	B	B	4.5	3.6
CCR09_F2-124	8	B	B	5.3	3.6
CCR09_F2-130	8	B	B	4.9	2.7
CCR09_F2-145	8	B	B	4.3	3.6
CCR09_F2-183	8	B	B	5.6	4.3
CCR09_F2-190	8	B	B	6.0	4.1
CCR09_F2-210	8	B	J	2.1	2.6
CCR09_F2-219	8	B	J	3.5	2.9
CCR09_F2-003	8	J	B	2.1	2.6
CCR09_F2-014	8	J	B	1.9	2.5
CCR09_F2-141	8	J	B	3.5	2.3
CCR09_F2-148	8	J	B	3.1	2.7
CCR09_F2-156	8	J	B	3.9	2.5
CCR09_F2-001	8	J	J	0.9	1.5
CCR09_F2-020	8	J	J	1.9	2.1
CCR09_F2-029	8	J	J	1.8	2.3
CCR09_F2-043	8	J	J	1.3	2.0
CCR09_F2-075	8	J	J	1.5	1.6
CCR09_F2-181	8	J	J	1.6	1.9
CCR09_F2-191	8	J	J	1.4	1.9

[a]Genotype of each QTL: B, homozygous for susceptibility allele derived from TCS-B1 and J, homozygous for resistance allele from TCS-J1

Supplemental Table 4. High-resolution melting (HRM) marker information developed for this study

Marker name	Physical position[a]	Primer sequence (5'-3')[b]	Length (bp)	Type of mutation	Mutation site in Chinese long v2/v3	Amplicon (bp)	QTL name
HRM-01	16,965,758	TGGCTTTTCCTTTGCATTCTCA<br>TGTCAAACTAGAACCTTGAAAC	22<br>24	SNP	No gene	84	*Tls5.1*
HRM-02	24,473,862	CGCAGGCATAGAAGTATTTACG<br>TGGTCCAAACTTTCCCGGAA	23<br>20	SNP	Csa5G621930	97	
HRM-03	24,584,092	ATAAGGAGTGGGAGGAGAAGA<br>GAGAGCAAAACAGAGTTCAC	21<br>20	SNP	CsaV3_5G035980.1	83	
HRM-04	24,646,609	GGCCCAACCACGTGATAGTT<br>CCCAATCGTGTAAAACCAGCTG	20<br>22	SNP	Csa5G622660	89	
HRM-05	24,670,496	TCTGATAAAGCACGAAACTGCA<br>AGTTTTTGTTTTGTGCATTAAGT	22<br>26	SNP	Csa5G622700	80	
HRM-06	24,670,681	TGAACCGTATTCTCGACCAGC<br>AGTGTATGCAGTATTTAGTTCAT	21<br>27	SNP	Csa5G622700	130	
HRM-07	24,749,906	ACCTCTTTTGCCAGCCTTCT<br>GGTCACTATATGTATGGCTGTCC	20<br>24	SNP	Csa5G622840	122	*Tls5.2*
HRM-08	25,167,471	AGGTCGTGGTTTTTGAACTCC<br>TGACTCGACATGGGATTTTGGA	21<br>22	SNP	Csa5G624460	130	
HRM-09	25,674,360	GCGATGCTCACAAGACTTCC<br>TAAAAACTGCAGGTCAGCCT	20<br>20	SNP	Csa5G630800	70	
HRM-10	25,781,214	AATCGGAATTTTAACCTCGACG<br>TGGTCCAATGCAGCTCTTCA	22<br>20	SNP	Csa5G630970.1	129	
HRM-11	25,834,218	AGGAACCGTGGATGATTGCA<br>AGGTTGGACCCTGCTACTTA	20<br>20	SNP	No gene	130	
HRM-12	27,063,405	ACTACATGCAGAAGAACCACAC<br>AGGAACTCTTGGTCATCCAGC	22<br>24	SNP	Csa5G644530.1	120	

[a]Physical position in Chinese Long v2 reference genome

[b]For each marker, the top and bottom rows are the forward and reverse sequences, respectively

Supplemental Table 5. List of 69 genes annotated in the *Tls5.2* fine-mapped region

No.	Gene ID[a]	Description
1	Csa5G622790	Vacuolar protein sorting, putative; contains IPR001619 (Sec1-like protein), IPR027482 (Sec1-like, domain 2)
2	Csa5G622800	Transferase, transferring glycosyl groups, putative
3	Csa5G622810	BTB/POZ domain-containing protein; contains IPR011333 (BTB/POZ fold), IPR027356 (NPH3 domain)
4	Csa5G622820	Dolichyl-diphosphooligosaccharide-protein glycosyltransferase subunit; contains IPR007676 (Ribophorin I)
5	Csa5G622830	GATA transcription factor, putative; contains IPR016679 (Transcription factor, GATA, plant)
6	Csa5G622840	Probable zinc metalloprotease; contains IPR007484 (Peptidase M28)
7	Csa5G622850	Unknown protein
8	Csa5G622860	mRNA-decapping enzyme 2; contains IPR007722 (mRNA decapping protein 2, Box A), IPR015797 (NUDIX hydrolase domain-like)
9	Csa5G623360	Aquaporin 2; contains IPR000425 (Major intrinsic protein), IPR023271 (Aquaporin-like)
10	Csa5G623370	Unknown protein
11	Csa5G623380	Alpha,alpha-trehalose-phosphate synthase; contains IPR001830 (Glycosyl transferase, family 20), IPR003337 (Trehalose-phosphatase), IPR023214 (HAD-like domain)
12	Csa5G623390	WPP domain-associated protein
13	Csa5G623400	Intracellular protein transport protein USO1
14	Csa5G623410	Unknown protein
15	Csa5G623420	Patatin T5, putative; contains IPR016035 (Acyl transferase/acyl hydrolase/lysophospholipase
16	Csa5G623430	Anthranilate synthase, component II; contains IPR005802 (Para-aminobenzoate synthase, component I), IPR017926 (Glutamine amidotransferase)
17	Csa5G623440	Unknown protein
18	Csa5G623450	Ubiquinone biosynthesis protein ubiB; contains IPR011009 (Protein kinase-like domain)
19	Csa5G623460	Ubiquitin-conjugating enzyme E2 C, putative; contains IPR016135 (Ubiquitin-conjugating enzyme/RWD-like), IPR023313 (Ubiquitin-conjugating enzyme, active site)
20	Csa5G623470	MLO-like protein; contains IPR004326 (Mlo-related protein)
21	Csa5G623480	Tetraspanin-6; contains IPR018499 (Tetraspanin/Peripherin)
22	Csa5G623490	Serine carboxypeptidase-like protein; contains IPR001563 (Peptidase S10, serine carboxypeptidase)
23	Csa5G623500	Nuclear receptor corepressor; contains IPR009057 (Homeodomain-like)
24	Csa5G623510	IQ domain-containing protein; contains IPR000048 (IQ motif, EF-hand binding site)
25	Csa5G623520	Mitochondrial import inner membrane translocase subunit TIM14, putative; contains IPR001623 (DnaJ domain)
26	Csa5G623530	Mitochondrial carrier protein, putative; contains IPR002067 (Mitochondrial carrier protein), IPR011992 (EF-hand-like domain), IPR023395 (Mitochondrial carrier domain)
27	Csa5G623540	ATP synthase D chain, mitochondrial, putative; contains IPR008689 (ATPase, F0 complex, subunit D, mitochondrial)
28	Csa5G623550	Crossover junction endonuclease MUS81; contains IPR011335 (Restriction endonuclease type II-like)
29	Csa5G623560	DELLA domain GRAS family transcription factor GAI; contains IPR005202 (Transcription factor GRAS)
30	Csa5G623570	Unknown protein
31	Csa5G623580	Yae1 domain-containing protein; contains IPR019191 (Essential protein Yae1, N-terminal)
32	Csa5G623590	Intron-binding protein aquarius; contains IPR026300 (CWF11 family), IPR027417 (P-loop containing nucleoside triphosphate hydrolase)
33	Csa5G623600	Unknown protein
34	Csa5G623610	HIT zinc finger family protein, expressed; contains IPR007529 (Zinc finger, HIT-type)
35	Csa5G623620	Unknown protein
36	Csa5G623630	Early-responsive to dehydration protein-like; contains IPR003864 (Domain of unknown function DUF221)
37	Csa5G623640	Annexin
38	Csa5G623650	Repetitive proline-rich cell wall protein
39	Csa5G623660	Annexin; contains IPR001464 (Annexin)
40	Csa5G623670	Annexin; contains IPR001464 (Annexin)
41	Csa5G623680	Annexin; contains IPR001464 (Annexin)
42	Csa5G623690	Annexin; contains IPR001464 (Annexin
43	Csa5G623700	GTPase obg; contains IPR005225 (Small GTP-binding protein domain), IPR014100 (GTP-binding protein Obg/CgtA), IPR027417 (P-loop containing nucleoside triphosphate hydrolase)
44	Csa5G623710	Putative uncharacterized protein T14E10_70
45	Csa5G623720	F-box protein SKIP5; contains IPR001810 (F-box domain, cyclin-like), IPR011050 (Pectin lyase fold/virulence factor)

46	Csa5G623730	E3 ubiquitin ligase-like protein; contains IPR004162 (Seven-in-absentia protein, sina), IPR013083 (Zinc finger, RING/FYVE/PHD-type)
47	Csa5G623740	ARF-GAP domain 13; contains IPR001164 (Arf GTPase activating protein), IPR008973 (C2 calcium/lipid-binding domain, CaLB)
48	Csa5G623750	Glutaredoxin family protein; contains IPR012336 (Thioredoxin-like fold)
49	Csa5G623760	Cytochrome c oxidase assembly protein; contains IPR018793 (Cytochrome c oxidase assembly protein PET191)
50	Csa5G623770	DNA repair helicase rad5,16, putative; contains IPR000330 (SNF2-related), IPR001650 (Helicase, C-terminal), IPR001810 (F-box domain, cyclin-like), IPR011124 (Zinc finger, CW-type), IPR017907 (Zinc finger, RING-type, conserved site), IPR027417 (P-loop containing nucleoside triphosphate hydrolase)
51	Csa5G623780	Annexin; contains IPR001464 (Annexin)
52	Csa5G623790	Annexin; contains IPR001464 (Annexin)
53	Csa5G623800	Response regulator 5; contains IPR011006 (CheY-like superfamily)
54	Csa5G623810	Probable peptide/nitrate transporter; contains IPR000109 (Proton-dependent oligopeptide transporter family), IPR016196 (Major facilitator superfamily domain, general substrate transporter)
55	Csa5G623820	Beta-galactosidase, putative; contains IPR006101 (Glycoside hydrolase, family 2), IPR008979 (Galactose-binding domain-like), IPR011013 (Galactose mutarotase-like domain), IPR013812 (Glycoside hydrolase, family 2/20, immunoglobulin-like beta-sandwich domain), IPR017853 (Glycoside hydrolase, superfamily), IPR023230 (Glycoside hydrolase, family 2, conserved site), IPR023232 (Glycoside hydrolase, family 2, active site)
56	Csa5G623830	Amidohydrolase family protein; contains IPR005920 (Imidazolonepropionase), IPR013108 (Amidohydrolase 3)
57	Csa5G623840	Short internode related sequence; contains IPR007818 (Protein of unknown function DUF702)
58	Csa5G623850	Phosphatidylinositol-4-phosphate 5-kinase, putative; contains IPR023610 (Phosphatidylinositol-4-phosphate 5-kinase), IPR027483 (Phosphatidylinositol-4-phosphate 5-kinase, C-terminal), IPR027484 (Phosphatidylinositol-4-phosphate 5-kinase, N-terminal domain)
59	Csa5G623860	NADPH:quinone oxidoreductase, putative; contains IPR005025 (NADPH-dependent FMN reductase)
60	Csa5G623870	Aspartic proteinase nepenthesin-1, putative; contains IPR001461 (Peptidase A1), IPR021109 (Aspartic peptidase)
61	Csa5G623880	Transcription factor
62	Csa5G623890	Unknown protein
63	Csa5G623900	Zinc finger protein, putative; contains IPR013087 (Zinc finger C2H2-type/integrase DNA-binding domain)
64	Csa5G623910	Ankyrin-repeat containing protein, putative; contains IPR011009 (Protein kinase-like domain), IPR013083 (Zinc finger, RING/FYVE/PHD-type), IPR020683 (Ankyrin repeat-containing domain)
65	Csa5G623920	Sigma factor binding protein 1[b]
66	Csa5G624420	Unknown protein
67	Csa5G624430	BZIP transcription factor; contains IPR004827 (Basic-leucine zipper domain), IPR025422 (DOG1 domain)
68	Csa5G624450	Peptidyl-prolyl cis-trans isomerase; contains IPR002130 (Cyclophilin-like peptidyl-prolyl cis-trans isomerase domain), IPR024936 (Cyclophilin-type peptidyl-prolyl cis-trans isomerase)
69	Csa5G624440	NADH dehydrogenase [ubiquinone] 1 alpha subcomplex subunit 6

[a]Gene ID in Chinese Long v2 reference genome

[b]Gene description in Chinese Long v3 reference genome
